# A Modified Anastomosis Technique for Esophagojejunostomy after Laparoscopy-Assisted Total Gastrectomy: A Single Team Preliminary Experience

**DOI:** 10.1155/2022/4494401

**Published:** 2022-01-18

**Authors:** Zehui Wu, Bing Wang, Gang Liu, Jiaju Lu, Chengxiong Zhang, Fangzheng Chen, Lianghui Shi, Aman Xu

**Affiliations:** ^1^Department of General Surgery, The First Affiliated Hospital of Anhui Medical University, Hefei 230022, Anhui Province, China; ^2^Department of Gastrointestinal Surgery, The First Affiliated Hospital of Wannan Medical College, Wuhu241001, Anhui Province, China

## Abstract

**Results:**

There were no significant differences between the cRY group and pRY group regarding age, sex, BMI, neoadjuvant therapy, preoperative comorbidities, history of laparotomy, ASA score, tumor location, pathological stage, total operative time, incision length, blood loss, time-to-first flatus, time-to-first soft diet, and postoperative hospital stays. The proportions of patients who received a 21 mm stapler were higher in the cRY group (7/44) than that in the pRY group (0/68) (*P* = 0.003). 7 anastomotic complications were reported (6 in the cRY group versus 1 in pRY group; *P* = 0.028) of which five (83.3%) in the cRY were anastomotic stenosis versus none in the pRY group (*P* = 0.044).

**Conclusions:**

The application of pant-shaped anastomosis for esophagojejunostomy after LTG is a safe and feasible procedure and has an advantage when the jejunum diameter is small.

## 1. Introduction

With the advent and increasing experience of minimally invasive techniques and technology, laparoscopic total gastrectomy (LTG) has gradually gained popularity because of its minimal invasiveness compared with open total gastrectomy (OTG). However, postoperative anastomotic complications such as leakage and stenosis sometimes still occur. A recent meta-analysis showed that the incidence of anastomotic complications was slightly higher in LTG than in OTG [1]. Esophagojejunostomy is the most technically difficult type of anastomosis in the field of laparoscopic gastrectomies and associates with the risk of anastomotic leakage and stenosis [2, 3]. Anastomotic complications not only lower the survival rate but also affect the long-term quality of life [4]. So, attention should be paid to the choosing of an appropriate reconstruction method for esophagojejunostomy.

In the current status of LTG, most surgeons still prefer extracorporeal anastomosis through a minilaparotomy (laparoscopy-assisted total gastrectomy, LATG) rather than total laparoscopic intracorporeal anastomosis, which is more difficult. End-to-side Roux-en-Y esophagojejunostomy by using a circular stapler is widely accepted as a standard reconstruction method, with low rates of anastomotic leakage. However, although seldom reported, the difficulty of stapler insertion sometimes occurs during operation. An obvious difficulty is the insertion and fixation of the anvil head through a narrow window. By using the orally inserted anvil (OrVil™) or reverse puncture device technique, this problem has been solved to some extent [5, 6]. Another important but often overlooked issue is how to successfully insert the main body of the stapler when the jejunum diameter is small. If not handled properly, this may lead to significant anastomotic complications. Hunt-Lawrence pouch, a broad enteroanastomosis between the afferent and efferent portion of the jejunal loop used for the EJS, was originally constructed to increase reservoir capacity and delay evacuation [7, 8]. However, this procedure has not been widely reported in LTG. Recently, we have devised a modification to this procedure for esophagojejunostomy, which can make the insertion of the stapler easier, and we applied it in 68 cases of LATG. We named it “pant-shaped Roux-en-Y esophagojejunostomy.” This article is aimed at sharing our experience of this modified anastomosis technique for LATG and to compare it with conventional Roux-en-Y esophagojejunostomy.

## 2. Patients and Methods

### 2.1. Patients

**Figure 1:**
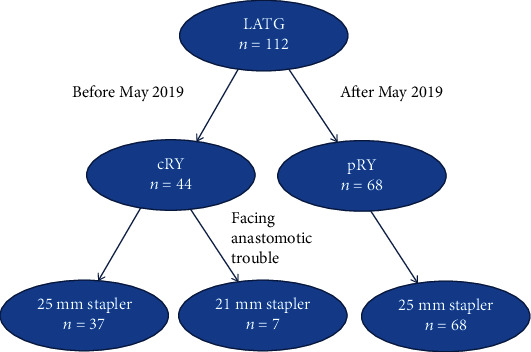
Flowchart of the study.

From January 2016 to December 2020, our team (led by L. Shi) performed 112 LATG with D2 lymph node dissection for middle-upper-third gastric cancer. Of these, 44 received a conventional Roux-en-Y end-to-side esophagojejunostomy, and the remaining 68 received pant-shaped Roux-en-Y esophagojejunostomy (Figure 1). All patients underwent diagnostic and preoperative staging work-up according to a standard protocol which includes upper digestive endoscopy with gastric biopsy and computed tomography of the abdomen and chest. Patients with distant metastases, para-aortic lymph node involvement, and/or pre- or intraoperative diagnosis of T4 lesions (i.e., local invasion of other organs, including spleen, pancreas, or peritoneum) were not included for LATG. All patients signed an informed consent form before surgery. This study was approved by the Ethics Committee of Yijishan Hospital at Wannan Medical College. The data retrospectively collected included the following: (1) patients' general clinical characteristics (age, sex, body mass index, neoadjuvant therapy, preoperative comorbidity, etc); (2) surgical results (total operative time, estimated intraoperative blood loss, size of circular stapler used in esophagojejunal anastomosis, and incision length); and (3) short-term outcomes within 12 months after surgery, including time-to-first flatus, time to liquid diet, length of postoperative hospital stay, and postoperative complications. Postoperative complications were classified according to the Clavien–Dindo scoring system [9].

### 2.2. General Procedure

Under general anesthesia, patients lay in the supine position, with the head higher than the feet (forming a 15-degree incline). The surgeon stood on the left side of the patient, the first assistant was on the right, and the camera operator stood between the legs of the patient. A 12 mm trocar was inserted through an infraumbilical incision. After pneumoperitoneum was achieved, two right 5 mm assistant ports and two left operator ports (5 mm lower and 12 mm upper) were inserted under laparoscopy. After inspection of the peritoneal cavity, the left lobe of the liver was lifted using a suture retraction technique [10]. The stomach was mobilized using an ultrasonic scalpel with D2 lymphadenectomy according to the Japanese Gastric Cancer Treatment Guidelines [11]. The duodenum bulb was transected just 1 cm below the pyloric ring intracorporeally using an endoscopic linear stapler (Echelon 60; Ethicon Endo-Surgery).

Once the stomach was completely mobilized, a 6-10 cm vertical midline incision was made in the upper abdomen, and the fully mobilized stomach was pulled out of the abdominal cavity. Then, the stomach was transected 1-2 cm proximal to the gastroesophageal junction. Choice of the circular stapler size, 25 mm or 21 mm, is based upon the esophageal and jejunal diameter. Commonly, a 25 mm anvil head (EEA Circular Stapler DST Series; Covidien) was inserted into the esophageal stump under direct vision, and a purse-string suture was tied around its central rod. Next, the jejunum was divided 20 cm distal to the Treitz ligament. The Roux-en-Y jejunojejunostomy was created using a continuous 3-0 Vicryl suture (Ethicon, Cincinnati, USA), approximately 50 cm distal to the site of future esophagojejunostomy extracorporeally.

### 2.3. Conventional Roux-En-Y Anastomosis

The main unit of the 25 mm circular stapler (EEA Circular Stapler DST Series; Covidien) is inserted through the open jejunal end of the Roux limb with the central rod penetrating the antimesenteric wall 5 cm from the end of the loop. The anvil and circular stapler body were connected, and an end-to-side esophagojejunostomy was conducted under direct view. The instrument is then withdrawn and the open end of the jejunal loop is closed with a linear stapler (Echelon 60; Ethicon Endo-Surgery).

### 2.4. Pant-Shaped Roux-en-Y Anastomosis

**Figure 2:**
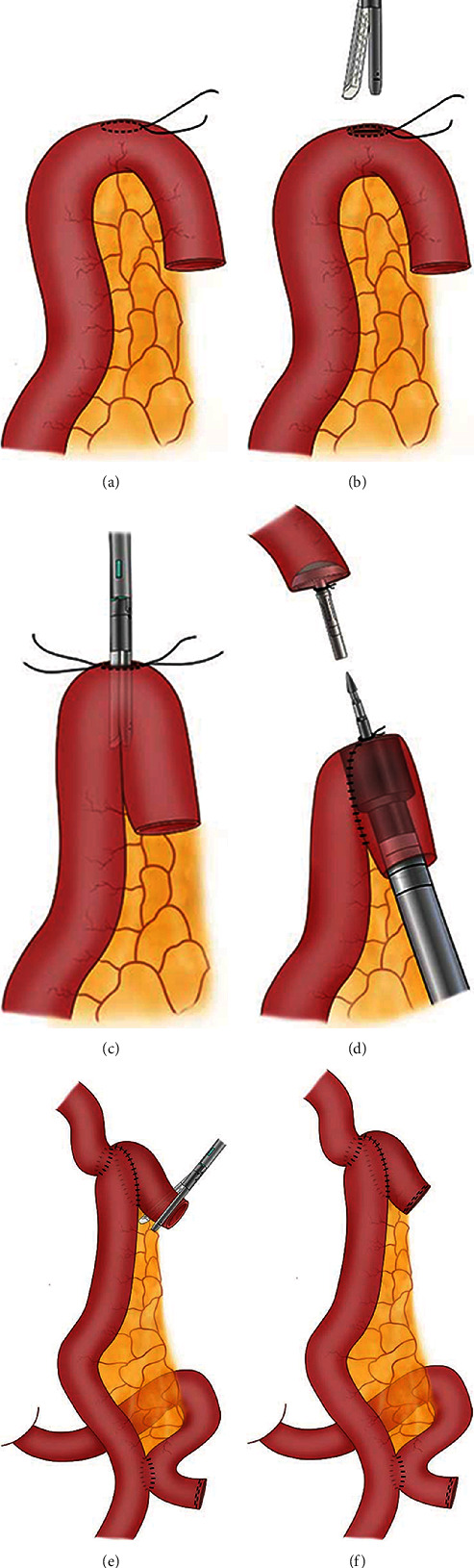
Schematic diagram of pant-shaped anastomosis. A purse-string suture was made on the antimesenteric edge 10 cm away from the jejunal end (a), followed by an incision in its center (b). The two arms of a linear stapler were inserted into the afferent and efferent portion of the jejunum, respectively (c). A circular stapler was inserted into the jejunal pouch through the jejunal end, with the central rod puncturing through the antimesenteric incision (d). After the EJS was finished, the remnant entry was closed using a linear stapler (e), which left the final appearance of the anastomosis similar to a pair of low crotch pants (f).

**Figure 3:**
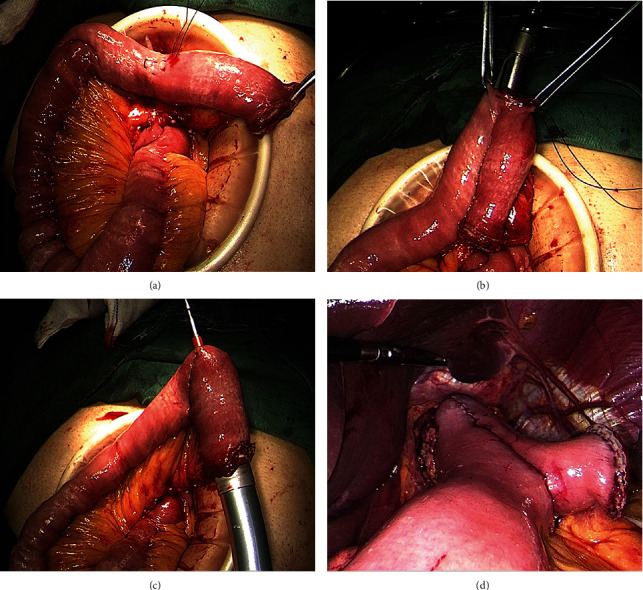
Surgical diagram of pant-shaped anastomosis. A purse-string suture was made on the antimesenteric edge 10 cm away from the jejunal end, followed by an incision in its center (a). The two arms of a linear stapler were inserted into the afferent and efferent portion of the jejunum, respectively (b). A circular stapler was inserted into the jejunal pouch through the jejunal end, with the central rod puncturing through the antimesenteric incision (c). After the EJS was finished, the remnant entry was closed using a linear stapler (d).

First, the proximal end of the Roux limb was folded on itself in a form similar to an “inverted J,” 10 cm long. A 2 cm enterotomy was made at the top corner of the folded jejunum loop with a purse-string suture around it (Figures 2(a) and 3(a)). Then, through this incision, we inserted the two arms of a linear stapler (Echelon 60; Ethicon Endo-Surgery) into the afferent and efferent portion of the jejunum loop in an up-to-bottom approach, respectively (Figures 2(b), 2(c), and 3(b)). By firing the stapler, a jejunal pouch was created. Next, the main unit of a 25-mm circular stapler was inserted from the end of the jejunum to the pouch, with the central rod puncturing through the incision at the top corner (Figures 2(d) and 3(c)). After that, the 25 mm circular stapler was introduced into the abdominal cavity. The anvil and circular stapler body were connected, and end-to-side esophagojejunostomy was conducted under direct view. Finally, the remnant entry hole was closed using a linear stapler (Figures 2(e), 2(f), and 3(d)). The final appearance of the esophagojejunal anastomosis looks like a pair of low crotch pants; so, we named it pant-shaped Roux-en-Y anastomosis.

### 2.5. Postoperative Care and Follow-Up

A nasojejunal tube is placed routinely and early enteric nutrition started within 24-48 hours postoperatively. Sips of water were allowed on the 2nd postoperative day, and a fluid diet was usually permitted on the 5th. All patients underwent routine water-soluble contrast swallow X-ray before restarting oral nutrition. The patients are usually discharged on the 11-14^th^ postoperative day. All participants were postoperatively followed up at the outpatient clinic at 1, 3, 6, and 12 months after discharge. Stenosis at the site of the esophagojejunostomy was suspected when patients reported dysphagia, and endoscopic examination was ordered. Stenosis was diagnosed during endoscopic examination when the endoscope could not pass through the anastomosis. All the patients without symptoms were examined 1 year after surgery for direct observation of the anastomosis site.

### 2.6. Statistical Analysis

Statistical analysis was performed with SPSS 26.0 software (SPSS Inc., Chicago, IL, USA). Continuous data are presented as mean ± standard deviation (SD) or median (range), and categorical data are presented as number (%). Student's *t*-test or Mann–Whitney *U* test was used for comparing continuous data, and the chi-square test or Fisher's exact test was adopted for comparing categorical data. *P* values <0.05 indicated statistical significance.

## 3. Results

### 3.1. Patient Characteristics

A total of 112 patients underwent successful LATG. Of these, 44 patients (29 men; 15 women) received a conventional Roux-en-Y end-to-side esophagojejunostomy (cRY group), and the remaining 68 patients (41 men; 27 women) underwent pant-shaped Roux-en-Y esophagojejunostomy (pRY group). The detailed information of these patients is shown in Table 1. There were no significant differences between the cRY group and the pRY group in terms of age, sex, BMI, neoadjuvant therapy, preoperative comorbidities, history of laparotomy, and ASA score. Tumor locations and pathological stages did not have any significant difference between the two groups.

### 3.2. Surgical Outcomes

The surgical outcomes and postoperative complications within 12 months after surgery are summarized in Table 2. There was no significant difference in total operative time, incision length, and blood loss between the cRY group and the pRY group. Besides, there was no significant difference in the time-to-first flatus, time-to-first soft diet, and postoperative hospital stays between the two groups. The proportions of patients who received a 21 mm stapler were higher in the cRY group (7/44) than that in the pRY (0/68) (*P* < 0.05). The seven cases choosing a 21 mm circular stapler for an esophagojejunostomy were all due to anastomotic trouble caused by the narrow diameter of the jejunum.

### 3.3. Postoperative Complications

There was no significant difference in incision complications and pulmonary complications between the two groups (Table 3). At an average follow-up time of 1.5 years, 7 anastomotic complications were observed (6 in the cRY group versus 1 in the pRY group; *P* = 0.028) of which four (66.7%) in the cRY were anastomotic stenosis versus none in the pRY group (*P* = 0.044, Table 3). No anastomotic bleeding was observed. Data for overall anastomotic complications and characteristics are listed in Table 4. Staplers used in cases 1, 2, 3, and 5 were all temporarily changed to 21 mm stapler due to the mismatch of 25 mm stapler during operation. In case 4, although 25 mm stapler was used, the jejunum were not large in diameter enough and inserting process was challenging. Cases 6 and 7 both received 25 mm stapler without special event during operation. Anastomotic leakage was observed in cases 5, 6, and 7 one week after operation. Case 5 was successfully treated by conservative treatment, including abdominal drainage, enteral nutrition, and antibiotics. In cases 6 and 7, reoperations were carried out due to failure of drainage. Anastomotic stenosis with early dysphagia was experienced by four patients (cases 1, 2, 3, and 4) who were addressed with several times of endoscopic balloon dilation.

## 4. Discussion

The first LTG for gastric cancer was reported by Azagra et al. in 1999 [12]. Compared with laparoscopic distal gastrectomy, LTG has not been adopted widely over the next twenty years because of the perceived complexity of the procedure. Esophagojejunostomy is the critical step with high technical demand during LTG [13]. Although various methods have been reported for esophagojejunostomy after LTG, there is no optimal EJS technique well recognized by scholars. To our knowledge, circular stapler-based extracorporeal reconstruction after LTG was still favored by many gastric surgeons, especially in the early phase of laparoscopic gastrectomy. The reason is that all reconstruction steps are almost the same as those in OTG except for a smaller abdominal incision. Difficulties mainly exist in the insertion and fixation of the anvil head through a narrow window. By using the transorally inserted anvil (OrVil™) or reverse puncture device technique, this problem has been solved to some extent [5, 6]. However, there are still some other drawbacks. One problem is that the Roux limb sometimes does not match the circular stapler well. When the diameter of the Roux limb is not large enough, a smaller (usually 21 mm) circular stapler will be chosen. However, Alasfar et al. reported a stenosis incidence of 23% after laparoscopic Roux-en-Y gastric bypass using a 21 mm circular stapler [14]. Fukagawa et al. showed that the use of a circular stapler with a smaller size (21 mm) significantly increased the rate of anastomotic stenosis [15]. Moreover, Zuiki et al. reported that the use of 21 mm EEA stapler is an independent risk factor for the development of anastomotic stenosis [2]. These studies are consistent with our previous experience; so, we preferred 25 mm circular stapler for esophagojejunostomy in practice. In the present study, among 44 cases with traditional Roux-en-Y anastomosis, 39 cases chose 25 mm stapler, and 7 cases encountered anastomotic troubles caused by the narrow diameter of jejunum; so, they finally chose 21 mm stapler. Unfortunately, anastomotic complications eventually occurred in 4 of the 7 cases (1 anastomotic leakage and 3 anastomotic stenoses).

The Hunt-Lawrence pouch is a broad enteroanastomosis between the afferent and efferent portion of the jejunal loop used for the EJS. It was first described in 1952 and aims to create a “neo-reservoir” to reduce nutrition-related complications [7]. With the use of an autosuture stapler, the procedure of Hunt-Lawrence pouch reconstruction during total gastrectomy becomes simple [16]. First, the proximal end of the Roux limb was folded on itself, and two small enterotomies were made distally on two limbs. Then, a linear stapler was inserted in a bottom-to-up approach to perform side-to-side enteroanastomosis. After that, the end-to-side EJS was performed with a circular stapler through the same orifice used for the linear stapler. Finally, the common enterotomy was closed. Since the pouch is wide enough, a large circular stapler can be easily introduced, and the “neo-reservoir” allowed more postoperative food intake. Studies showed that Hunt-Lawrence pouch reconstruction after total gastrectomy was superior to the conventional Roux-en-Y esophagojejunostomy for improved dietary intake and quality of life [17, 18]. However, this procedure has not been widely reported in LTG. We applied Hunt-Lawrence pouch reconstruction during LTG in several cases by using the method reported by Ward et al. [19] and found two potential drawbacks. One problem is that a mucosa bridge usually remains at the top of the pouch and cannot be completely cut by the head of the linear stapler. Another problem is that the common entry of the limbs had to be closed with hand sewn. So, we made a small modification to the procedure by creating an enterotomy at the top corner of the folded jejunum loop instead of two enterotomies in the limbs. Through this incision, a linear stapler was inserted into the jejunum in an up-to-bottom approach. With this modification, all anastomosis can be performed by using an autosuture stapler, and no hand sewn is needed. Because the final shape of the anastomosis is quite similar to a pair of pants (esophagojejunal anastomosis as pants waist, enteroanastomosis as pants crotch, jejunum limbs as legs), we named this procedure pant-shaped anastomosis.

We first used this modified method for esophagojejunal anastomosis in May 2019. According to our experience, there are several advantages of pant-shaped anastomosis. First, this reconstruction method is not limited by the intestinal diameter, thus reducing the risk of anastomotic stenosis. In pRY anastomosis, the intestinal blind loop is commonly left short. Since the pouch is large enough, the only factor limiting the insertion of the stapler is the diameter of the small intestine's end orifice. When the diameter of intestinal end is smaller than that of circular stapler, the antimesenteric wall near the end can be cut for 0.5 to 1 cm to increase the opening diameter of the intestine and allow the head of the circular staplerto pass easily. This is why all patients in the pRY group received 25 mm circular staplers successfully. Second, all anastomosis can be made by using autosuture staplers without hand-sewn in a narrow and deep operative field. Therefore, this procedure is very simple and easy for surgeon. Third, because the enteroanastomosis was performed at the antimesenteric edge, the blood supply will not be damaged, and the risk of anastomotic leakage will not be increased. Also, the increased reservoir capacity may potentially improve the patient's postoperative quality of life in the long-term (this requires the jejunal pouch to be large enough). Our initial purpose was to investigate the feasibility and efficacy of pant-shaped anastomosis in esophagojejunostomy in LATG; so, we only applied a charge of 60 mm length linear stapler cartridge to construct a small jejunal pouch. Thus, the reservoir effect is small, and it was not in the scope of this study.

By December 2020, our team had performed 68 LATGs with pant-shaped Roux-en-Y anastomosis. Compared with our previous 44 LATG with conventional Roux-en-Y anastomosis, the present study showed that pant-shaped Roux-en-Y was feasible and safe in terms of operative time, intraoperative blood loss, and overall postoperative complications. As to the incidence of anastomotic complications, the pRY group was lower than that in the cRY group. In the pRY group, all esophagojejunostomies were successfully performed with a 25 mm circular stapler. In the cRY group, a certain proportion (7/68) of patients could only receive 21 mm stapler. Our results showed that the rate of anastomotic complications, particularly stenosis, is relatively high in cases where 21 mm staples were used (leakage: 1/7; stenosis: 4/7). In Zuiki's study, stenosis occurred in 3 (60%) of 5 patients when the 21 mm end-to-end anastomosis stapler, which is close to our results. We think that this may contribute to the high incidence of anastomotic complications in the cRY group. First, using a 21 mm stapler itself leads to a smaller anastomotic diameter. Second, repeated attempts to insert a mismatched stapler, sometimes with violent operation, inadvertently affect the integrity of the intestinal mucosa at the anastomosis site.

Although several methods for side-to-side anastomosis using linear stapler have been reported to reduce anastomotic stenosis, such as functional end-to-end anastomosis, the overlap method, and the *π*-shaped method, the rates of anastomotic leakage still exist [20–22]. More importantly, these methods are mainly suitable for patients with lesions in the body and fundus of the stomach as well as the lower cardia. For patients with lesions in the upper and middle cardia, esophagojejunostomy using a circular stapler is more desirable to ensure a negative surgical margin [23].

## 5. Conclusion

The application of pant-shaped anastomosis for digestive tract reconstruction after LATG proved to be safe and feasible. When the diameter of the jejunum is small, this method is simple and has an advantage over conventional Roux-en-Y esophagojejunostomy. This study provides a new method for laparoscopic gastrointestinal reconstruction.

## Figures and Tables

**Table 1:** Clinicopathological information of the patients in the cRY group and the pRY group.

Variable	cRY group (*n* = 44)	pRY group (*n* = 68)	*P* value
Age (years)	62.3 ± 9.1	65.2 ± 7.9	0.238
Sex(M/F)	29/15	41/27	0.468
BMI (kg/m^2^)	24.5 ± 2.2	24.0 ± 2.1	0.197
Neoadjuvant therapy	5 (11.4%)	9 (13.2%)	0.770
Preoperative comorbidities (total)	16 (36.4%)	22 (32.3%)	0.662
Hypertension	7 (15.9%)	10 (14.7%)	0.862
Diabetes mellitus	4 (9.1%)	5 (7.4%)	1.000
Heart disease	1 (2.3%)	2 (2.9%)	1.000
Chronic lung disease	2 (4.5%)	3 (4.4%)	1.000
Other comorbidities	2 (4.5%)	2 (2.9%)	1.000
History of laparotomy	6 (13.6%)	8 (11.8%)	0.770
ASA score (I:II:III)	25: 17: 2	35: 30 : 3	0.846
Tumor location			0.719
EGJ	27	44	
Gastric body	17	24	
TNM stage^a^ (I:II:III)	10: 20: 14	7: 38: 23	0.191

Values are presented as mean ± standard deviation, number, or number (%). ASA score: American Society of Anesthesiologists score; BMI: body mass index; EGJ: esophagogastric junction. ^a^TNM stage was evaluated according to the 8th edition of the AJCC Staging Manual.

**Table 2:** Surgical outcomes in the cRY group and the pRY group.

Variable	cRY group (*n* = 44)	pRY group (*n* = 68)	*P* value
Total operative time (min)	212.5 ± 16.4	213.1 ± 17.5	0.859
Operative blood loss (ml)	39.9 ± 21.0	39.6 ± 15.2	0.243
Incision length (cm)	6.9 ± 0.3	7.0 ± 0.2	0.585
Size of stapler			0.003^∗^
25 mm	37	68	
21 mm	7	0	
Time-to-first flatus	3.1 ± 0.3	3.2 ± 0.4	0.309
Time to liquid diet	4.5 ± 0.5	4.4 ± 0.5	0.380
Postoperative hospital stay (days)	12.8 ± 6.4	11.8 ± 3.5	0.343

Values are presented as mean ± standard deviation, number, or number (%). ∗Statistically significant.

**Table 3:** Postoperative complications in the cRY group and the pRY group.

Postoperative complications	cRY group (*n* = 44)	pRY group (*n* = 68)	*P* value
Incision complication	3 (6.8%)	4 (5.9%)	1.000
Postoperative bleeding	1 (2.3%)	1 (1.5%)	1.000
Pulmonary complication	1 (2.3%)	1 (1.5%)	1.000
Anastomotic complications	6 (13.6%)	1 (1.5%)	0.028^∗^
Anastomotic leakage	2 (4.5%)	1 (1.5%)	0.700
Anastomotic stenosis	4 (9.1%)	0	0.044^∗^
Anastomotic bleeding	0	0	—

Values are presented as number or number (%). ^∗^Statistically significant.

**Table 4:** The characteristics of cases with anastomotic complications.

Case number	Anastomotic complication	Anastomosis method	Stapler size	Treatment
1	Stenosis	cRY	21 mm	Endoscopic balloon dilation
2	Stenosis	cRY	21 mm	Endoscopic balloon dilation
3	Stenosis	cRY	21 mm	Endoscopic balloon dilation
4	Stenosis	cRY	25 mm	Endoscopic balloon dilation
5	Leakage	cRY	21 mm	Conservative treatment
6	Leakage	cRY	25 mm	Surgical intervention
7	Leakage	pRY	25 mm	Conservative treatment

## Data Availability

Data and materials would be made available upon request.
